# Huge malignant phyllodes breast tumor: a real entity in a new era of early breast cancer

**DOI:** 10.1186/s12957-015-0508-7

**Published:** 2015-02-27

**Authors:** Alberto Testori, Stefano Meroni, Valentina Errico, Roberto Travaglini, Emanuele Voulaz, Marco Alloisio

**Affiliations:** Department of Thoracic and General Surgery, Humanitas Research Hospital, Via Manzoni, 56, Rozzano, Milan Italy; Division of Breast Radiology, European Institute of Oncology, Via Ripamonti, 435, Milan, Italy

**Keywords:** Phyllodes breast tumor, Breast cancer, Axillary metastasis

## Abstract

Phyllodes tumor is an extremely rare tumor of the breast. It occurs in females in the third and fourth decades. The difficulty in distinguishing between phyllodes tumors and benign fibroadenoma may lead to misdiagnosis. Lymph node involvement is rarely described in phyllodes tumors; for this reason, sentinel node biopsy may be warranted. We present a case of a 33-year-old woman affected by huge tumor of the right breast with ulceration in the skin with a rapid tumor growth and with omolateral axillary metastasis.

## Background

Malignant phyllodes tumor is a rare lesion of the breast that can mimic breast benign mass on clinical diagnosis; on the other hand, it is characterized by a typical rapid growth. Clinicians and breast radiologists should pay particular attention to these features, in order to avoid a delayed diagnosis resulting in an aggressive surgical approach. In particular, breast radiologists could take into consideration ultrasound abnormalities in breast nodules and could accurately evaluate axillary lymph nodes in case of a breast lesion with rapid tumor growth. Here we present a case of a woman affected by a phyllodes tumor, an uncommon oncologic disease.

### Introduction

Phyllodes tumors have an incidence of 1 per 100,000 women and account for only 0.5% of all breast neoplasms [1]. They have been variously classified, such as ‘cystosarcoma phyllodes’.

The phyllodes tumor is classified into benign, malignant, and borderline tumor according to histopathological features [2,3]; its clinical course is frequently unpredictable. The malignant phyllodes tumor is rare with a lower incidence than the benign counterpart. The tumor usually occurs in 35- to 55-year-old women [4,5].

The tumor appears clinically as a round, mobile, and painless mass and there is no clinical features to distinguish benign or malignant phyllodes tumors from benign lesions [6]. This is the case of small-sized phyllodes tumors that could not be distinguished from fibroadenomas. There are no pathognomonic mammographic or ultrasound features [7]. So, pseudo angiomatous stromal hyperplasia (PASH), fibroadenoma, intraductal papilloma, or complex cyst are the main differential diagnoses. Rare malignant tumors, including medullary or mucinous carcinoma, may be considered as well. Even though imaging plays a fundamental role as a first diagnostic approach, final diagnosis is confirmed only by histological examination and immunohistochemical analysis.

Surgical resection remains the gold standard of treatment, whereas radiation therapy and chemotherapy have an undefined role.

The incidence of local recurrence, distant metastasis, and cancer-related death are relatively lower than previously reported [8].

In this case report, we discuss a patient who developed a rapidly expanding malignant phyllodes breast tumor with a diameter of about 40 cm and with omolateral axillary lymph node metastasis.

## Case report

We report a case of a 33-year-old female with no history of familiar huge phyllodes breast tumor or breast and ovarian cancer.

The patient had a small lump in the right breast, which appeared 4 years earlier during pregnancy and misinterpreted as mastitis.

The lump grew up gradually. She did not feel any pain or discomfort. In the recent months, the lump grew rapidly and breast skin appeared dark in color.

**Figure 1:**
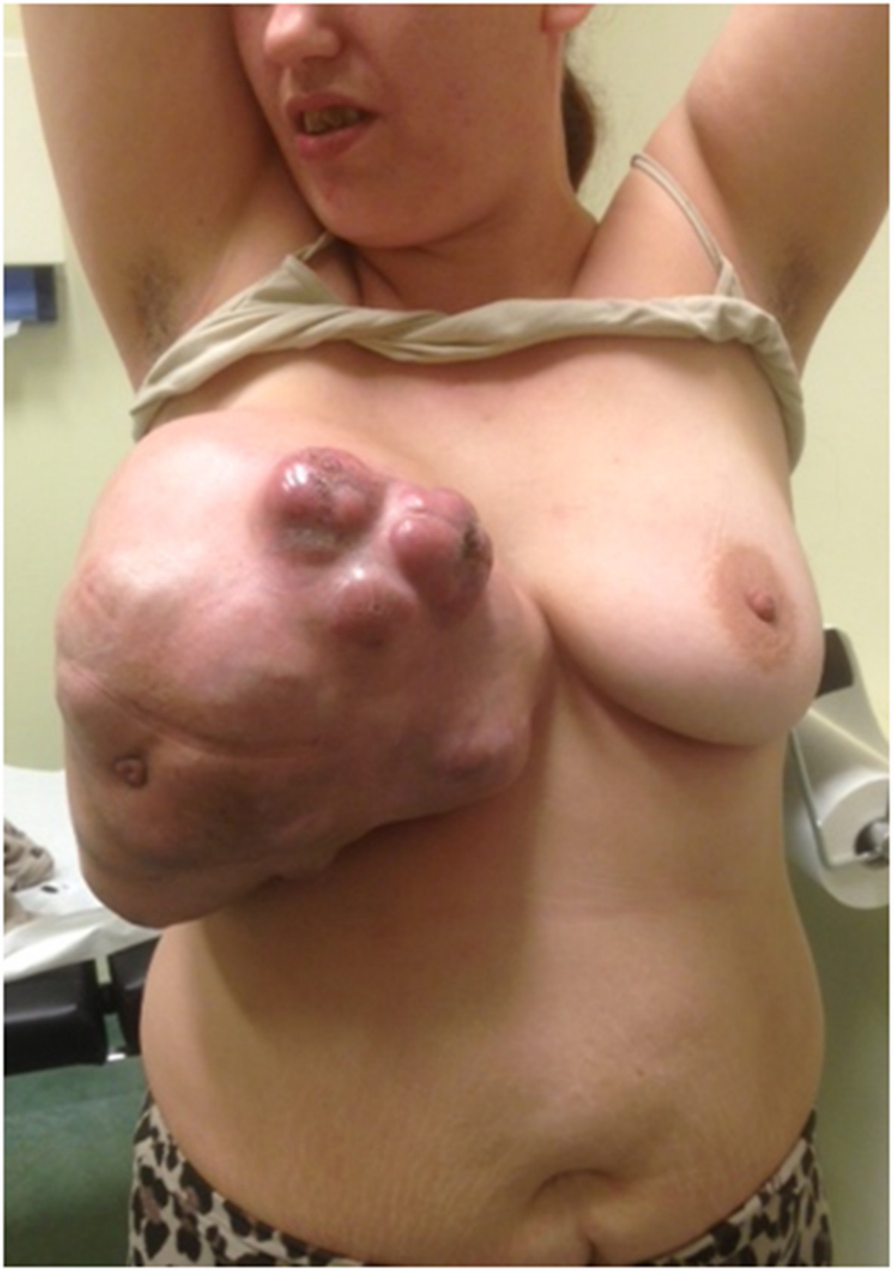
**Malignant phyllodes tumor of the right breast with a diameter of 40 cm in a 33-year-old patient.** The visible vessels, the thinned skin, and ulceration through the skin with exudate and foul smell are characteristics for large phyllodes tumors.

Physical examination revealed an enlarged right breast completely subverted by the neoplastic mass. It was 40 cm × 30 cm × 10 cm in size. Figure 1 shows the thin skin of right breast and the engorgement of right breast superficial vein. No axillary lymph node was palpable. The examination of the left breast was normal.

Ultrasonography showed a nodular blood flow in the heterogeneous lesion. These tumors can present cystic echoes, generally due to hemorrhage, necrosis, and mucoid degeneration.

In order to prevent bleeding or infections, we decided not to perform any interventional procedures for a preoperative diagnosis, considering the heterogeneous ultrasonographic features.

Due to the large size of the tumor and to the surgical indication for a total mastectomy, we did not perform mammography.

A preoperative positron emission tomography (PET/CT) scan was performed and showed a massive area of pathological accumulation of the tracer ^18^ F-FDG (^18^ F-Fludeoxyglucose) in the right breast suspected for cancer. The photopenic portion of the lesion could be a necrotic process. Right axillary lymph nodes showed a rapid volume increase and a pathologic accumulation of the tracer.

Skeletal and lung X-rays were normal. Biochemical examination revealed no significant abnormalities.

**Figure 2:**
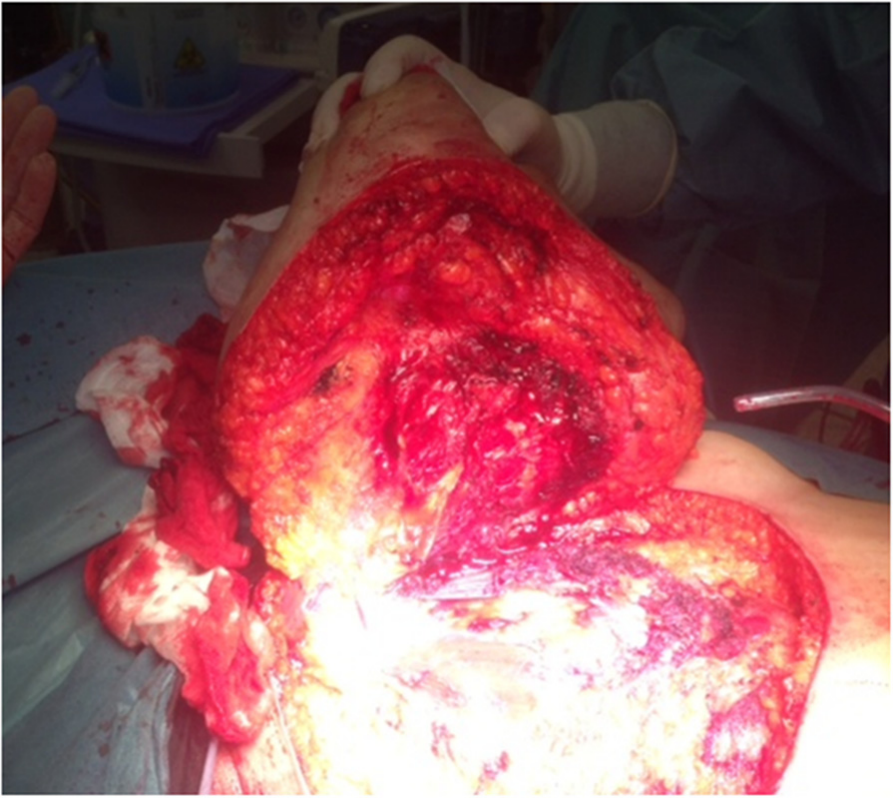
**Operative finding during radical mastectomy with partial resection of major pectoral muscle.**

The right breast and major pectoral muscle were excised with omolateral axillary lymphadenectomy (Figure 2). The resected specimen was a huge mass; the tumor did not invade surrounding tissues.

The histopathological examination showed a tumor with stromal hypercellularity and the presence of benign glandular elements; the margins of the specimen were free of disease.

HE staining and immunohistochemistry results were as follows: mitotic rates of 12 mitosis/10 high power fields (HPF), Desmin (−), Vimentin (+), Ck Pool (−), Ki67 (25-30%), SMA (+), S100 (−), CD117 (−), CD34 (+/−). The final diagnosis was malignant phyllodes tumor with metastasis in one out of nine lymph nodes.

Postoperative course was uneventful and the patient was discharged on the third postoperative day. Adjuvant chemotherapy (adriamicyn and ifosfamide) and adiuvant radiotherapy were administrated. Treatment was well tolerated and the patient was symptom-free at 18-month follow-up. Clinical and ultrasound examination were performed every 6 months. We recommended mammography every year for the surveillance of contralateral breast.

### Discussion

The phyllodes tumor of the breast is a rare disease usually presents as a large lump. In few cases, it is bilateral or multifocal [1,3,5,6,8]. It occurs mainly in middle-aged women. As reported in literature, mean age ranges from 30 to 52 years. Phyllodes tumors of the breast are commonly classified as benign tumors and rarely as borderline or malignant tumors [2,3,9-11].

The low incidence of phyllodes tumors could explain why the percentage of malignant phyllodes tumors reported in literature varies from 8% to 45% [12].

The difficulty in distinguishing between phyllodes tumors and benign fibroadenoma may lead to misdiagnosis. In fact, there are no characteristic features that clinically distinguish phyllodes tumors from other breast tumors.

Surgical treatment is generally the treatment of choice for phyllodes tumors, regardless of its histological subtype. Most studies recommend a more than 1- to 2-cm excision margin [4,5,13-16] based on the evidence that local recurrence occurs more frequently in patients with narrow surgical margins less than 1 to 2 cm.

However, an excision with the required margins is often impossible in huge phyllodes tumors due to the narrow area of breast tissue surrounding the lump.

Lymph node involvement is rarely described in phyllodes tumors [9-11]; so, routine axillary lymph node dissection is often unnecessary [8]. The most common path is the hematogenous spread, which occurs mostly in the lungs, pleura, and bones, such as in sarcoma [17].

Based on the available clinical data [8], little is known about malignant breast phyllodes tumors with positive axillary lymph node. Nodal metastases are rare in these patients; for this reason, sentinel node biopsy may be warranted. Formal axillary dissection could be unnecessary, but we think that the removal of low axillary lymph nodes (as in our case) may be recommended, especially in those patients with palpable lymphadenopathy or huge breast mass [18].

In our case, the preoperative PET/CT scan showed a suspicious lymph node in the right axilla. So, we think that some malignant breast phyllodes tumors can spread; sentinel lymph node biopsy may evaluate axillary involvement in these patients. It is difficult to make a correct preoperative evaluation in such cases but, in our opinion, the sentinel lymph node biopsy is a reasonable option.

The prognosis for malignant breast phyllodes tumors is poor and the role of various treatment modalities is not clearly defined due to the rarity of disease [19].

According to Ramakant *et al.* [9], large or giant phyllodes tumors (>10 cm) have higher cancer rates (42.5%) and recurrence rates (41%) compared with smaller tumors (21% malignancy rate and 29% recurrence rates). So, more aggressive treatments and adequate resection margins are needed. Radiation therapy is recommended in these selected cases, as in our case, and should be administrated within 4 months from surgery [6,19,20]. Multimodality postoperative treatment, such as adjuvant chemotherapy and radiotherapy, are recommended in tumors at high risk for local recurrence and metastatic spread, but their use in malignant phyllodes tumors is yet controversial.

Radiotherapy has been used with good results for local control of the disease [21] and it may be considered for high risk phyllodes tumors, including those greater than 5 cm, with stromal overgrowth, with more than 10 mitoses per HPF or with positive margins [6,19].

## Conclusions

Breast phyllodes tumor is a rare tumor. The early age of onset and the rapid growth make this report a special case.

The diagnosis was confirmed by pathology and immunohistochemistry. Surgical extended resection is the treatment of choice; we performed total mastectomy with adequate surgical margins and omolateral axillary lymph node dissection.

Adjuvant radiotherapy and chemotherapy may be administered in patients with high-grade tumors (as in our case), positive surgical margins, or postoperative recurrence, but their role is undefined.

In conclusion, we underline that phyllodes breast cancer may spread to distant organs and to axillary lymph nodes. Ultrasound abnormalities in breast nodules and breast lesions with atypical clinical and radiological presentations may be taken into particular consideration.

## Consent

Written informed consent was obtained from the patient for publication of this Case report and by accompanying images. A copy of the written consent is available for review by the Editor-in-Chief of this journal.
